# Testing alertness of Helicopter Emergency Medical Service (HEMS) crews – a feasibility study

**DOI:** 10.1186/1757-7241-22-S1-O1

**Published:** 2014-07-07

**Authors:** Teresa Cullip, Anthony Hudson, Richard Lyon, Emily McWhirter

**Affiliations:** 1 Kent, Sussex and Surrey Air Ambulance Trust, Kent, UK

## Background

In-shift alertness is a critical factor in patient safety. Previous studies have shown that whilst subjective fatigue in HEMS crews worsens over time, tests show no reduction in alertness. This project aimed to measure in-shift alertness levels amongst HEMS clinical crew members during day shifts at Kent, Sussex and Surrey Air Ambulance Trust.

## Method

Research was carried out across seven 12-hour day shifts. Background data and in-shift activities were recorded for each crew member to determine if these factors correlated with alertness levels. Data recorded included age, gender, job title, years of experience, hours of sleep pre-shift and number of shifts worked in previous 3 days. In-shift data was recorded at each test, including activities since last test, such as job deployment or kit checking, and whether food and drink had been consumed.

Alertness was measured every two hours using two computer based tests; the Stroop Colour Test (SCT), and the Psychomotor Vigilance Test (PVT).

## Results

It was not possible to collect data every two hours as planned due to the operational activities of the service. 64 test scores were collected over the course of 7 shifts. The test scores varied considerably, but showed an overall improvement in the SCT score over time, and no significant change in the PVT score. No correlation was found with any of the background factors measured, and in-shift activities such as job deployments did not significantly affect alertness levels.

## Conclusion / discussion

Alertness varies during a day shift, but this HEMS crew was able to maintain alertness despite reported fatigue. Background and in-shift activity did not seem to impact on crew alertness, but this warrants further study. In-shift testing is difficult to conduct due to operational activities.
